# Supplementary material for: Causal Relationship between Aging and Anorexia Nervosa: A White-Matter-Microstructure-Mediated Mendelian Randomization Analysis
Source: Biomedicines. 2024 Aug 16;12(8):1874. doi: 10.3390/biomedicines12081874 (PMC11351342; doi:10.3390/biomedicines12081874)
Supplement: Supplementary file 1 [file biomedicines-12-01874-s001.zip › Supplementary Figure/Figure S4/Singe SNP plot of MR analysis for TL to ubm-b-1868.pdf]

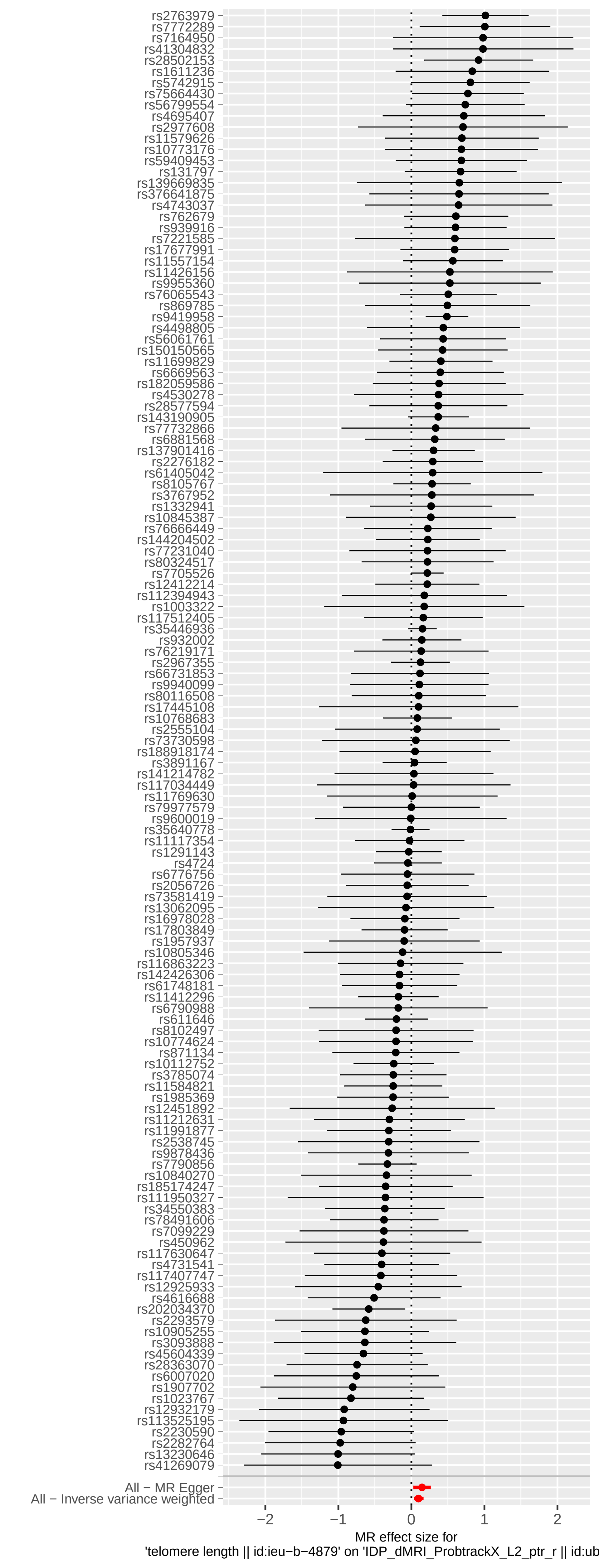
rs2763979
rs7772289
rs7164950
rs41304832
rs28502153
rs1611236
rs5742915
rs75664430
rs56799554
rs4695407
rs2977608
rs11579626
rs10773176
rs59409453
rs131797
rs139669835
rs376641875
rs4743037
rs762679
rs939916
rs7221585
rs17677991
rs11557154
rs11426156
rs9955360
rs76065543
rs869785
rs9419958
rs4498805
rs56061761
rs150150565
rs11699829
rs6669563
rs182059586
rs4530278
rs28577594
rs143190905
rs77732866
rs6881568
rs137901416
rs2276182
rs61405042
rs8105767
rs3767952
rs1332941
rs10845387
rs76666449
rs144204502
rs77231040
rs80324517
rs7705526
rs12412214
rs112394943
rs1003322
rs117512405
rs35446936
rs932002
rs76219171
rs2967355
rs66731853
rs9940099
rs80116508
rs17445108
rs10768683
rs2555104
rs73730598
rs188918174
rs3891167
rs141214782
rs117034449
rs11769630
rs79977579
rs9600019
rs35640778
rs11117354
rs1291143
rs4724
rs6776756
rs2056726
rs73581419
rs13062095
rs16978028
rs17803849
rs1957937
rs10805346
rs116863223
rs142426306
rs61748181
rs11412296
rs6790988
rs611646
rs8102497
rs10774624
rs871134
rs10112752
rs3785074
rs11584821
rs1985369
rs12451892
rs11212631
rs11991877
rs2538745
rs9878436
rs7790856
rs10840270
rs185174247
rs111950327
rs34550383
rs78491606
rs7099229
rs450962
rs117630647
rs4731541
rs117407747
rs12925933
rs4616688
rs202034370
rs2293579
rs10905255
rs3093888
rs45604339
rs28363070
rs6007020
rs1907702
rs1023767
rs12932179
rs113525195
rs2230590
rs2282764
rs13230646
rs41269079
All − MR Egger
All − Inverse variance weighted
−2
−1
0
1
2
MR effect size for
'telomere length || id:ieu−b−4879' on 'IDP_dMRI_ProbtrackX_L2_ptr_r || id:ub
